# Pilot scale production of the vaccine adjuvant Proteoliposome derived Cochleates (AFCo1) from *Neisseria meningitidis* serogroup B

**DOI:** 10.1186/1471-2172-14-S1-S4

**Published:** 2013-02-25

**Authors:** Caridad Zayas, Domingo González, Reinaldo Acevedo, Judith del Campo, Miriam Lastre, Elizabeth González, Belkis Romeu, Maribel Cuello, Julio Balboa, Osmir Cabrera, Luisa Guilherme, Oliver Pérez

**Affiliations:** 1 Finlay Institute. Ave. 27 No. 19805, La Lisa. Havana, Cuba. AP. 16017, CP11600; 2 Heart Institute (InCor) of Sao Paulo, Brazil

## Abstract

The use of new adjuvants in vaccine formulations is a subject of current research. Only few parenteral adjuvants have been licensed. We have developed a mucosal and parenteral adjuvant known as AFCo1 (Adjuvant Finlay Cochleate 1, derived from proteoliposomes of *N. meningitidis* B) using a dialysis procedure to produce them on lab scale. The immunogenicity of the AFCo1 produced by dialysis has been already evaluated, but it was necessary to demonstrate the feasibility of a larger-scale manufacturing process. Therefore, we used a crossflow diafiltration system (CFS) that allows easy scale up to obtain large batches in an aseptic environment. The aim of this work was to produce AFCo1 on pilot scale, while conserving the adjuvant properties. The proteoliposomes (raw material) were resuspended in a buffer containing sodium deoxycholate and were transformed into AFCo1 under the action of a calcium forming buffer. The detergent was removed from the protein solution by diafiltration to a constant volume. In this CFS, we used a hollow fiber cartridge from Amicon (polysulfona cartridge of 10 kDa porosity, 1mm channel diameter of fiber and 0.45 m^2^ area of filtration), allowing production of a batch of up to 20 L. AFCo1 were successfully produced by tangential filtration to pilot scale. The batch passed preliminary stability tests. Nasal immunization of BALB/c mice, induced specific saliva IgA and serum IgG. The induction of Th1 responses were demonstrated by the induction of IgG2a, IFNγ and not IL-5. The adjuvant action over Neisseria (self) antigens and with co-administered (heterologous) antigens such as ovalbumin and a synthetic peptide from haemolytic *Streptococcus* B was also demonstrated.

## Background

Over the last decade, there has been a flurry of research on adjuvants for vaccines, and several novel adjuvants are now in licensed products or in late clinical stage development. The success of adjuvants in enhancing the immune response to recombinant antigens has led many researchers to re-focus their vaccine development programs. Successful vaccine development requires knowing which adjuvants to use and knowing how to formulate adjuvants and antigens to achieve stable, safe and immunogenic vaccines. In addition to the demonstration of safety, immunogenicity and protection in preclinical models (where a model exits) and clinical studies, any adjuvant system needs to demonstrate feasibility of large-scale manufacturing. This includes definition, selection, supply and characterization of the raw materials, as well as the development of production processes that will deliver a stable and consistent product. In order to reach the final product, manufacturing processes, analytical and immunological tools, and quality control testing need to be conducted successfully. Only through this strict and controlled process will the quality of future adjuvant vaccines be guaranteed [1]. In this work, we transformed Neisseria proteoliposomes into cochleate structures (AFCo1) using a cross flow filtration system (CFS). AFCo1 showed high stability, immunogenicity and adjuvant effect on heterologous co-administered antigens [2]. Obtaining AFCo1 by tangential filtration process would ensure the production of a large scale batch of a safe and immunogenic adjuvant.

## Materials and methods

### Raw materials and filtration

Proteoliposomes were prepared from the outer membranes of serogroup B *N. meningitidis* strain Cu 385-83 [3]. Cochleate structures were obtained from these proteoliposomes [4]. Briefly, AFCo1 formation was performed by diluting precipitated proteoliposomes with detergent. The detergent was eliminated and calcium buffer (CB), Tris 10mM and 5mM of calcium chloride, was incorporated using CFS. CFS was used in two steps: firstly, four washes with CB were carried out to form the cochleates and then the detergent from the protein solution was removed by diafiltration to a constant volume [5]. In this system, we used a hollow fiber from Amicon (polysulfona cartridge of 10 kDa porosity, 1 mm channel diameter of fiber and 0.45 m^2^ area of filtration), allowing us to obtain a batch of upto 20 L. Secondly, the excess calcium and other contaminants were eliminated by six diafiltration washes.

### Formulations and immunogenicity

AFCo1 formulation contains Neisseria protein 2 mg/ml in CB and thiomersal preservative at 0.05% (w/v). For co-administration studies ovalbumin (OVA) or a synthetic peptide from haemolytic *Streptococcus* B (PepVacTB1) were admixed with AFCo1. Each dose of this formulation contained 50µg of AFCo1 with 20 µg of OVA or 20 µg of PepVacTB1. Female BALB/c mice (6-8 weeks old, CENPALAB, Cuba) were immunized three times via the intranasal (i.n) route at 0, 7 and 14 days with 12.5 µL administered into each nostril. The sera and saliva were collected as previously described [2] . Serum and saliva antibody titers (IgG, IgA, and IgG subclasses) were determined by indirect ELISA [6]. The supernatants of spleen cell cultures were stimulated *in vitro* with *Neisseria* proteoliposomes and evaluated for cytokine production using ELISA kits from Pharmingen (San Diego, CA) according to the manufacturer’s instructions. The differences between the groups were evaluated by Graph Pad Prism 4 software.

### Preliminary studies of stability

The AFCo1 lots obtained were subjected to preliminary stability evaluation for 12 months. This study was carried out at a shelf temperature of 5 ± 3º C and stressed at 40° C. We also evaluated the chemical physical characteristics of the obtained AFCo1.

## Results

### CFS was employed to get large volume batches of AFCo1

Formation of AFCo1, in the first stage, was characterized by a constant diafiltration flow. However, during the washing steps (second stage) the flow had a tendency to increase with the increasing number of washes. The hydrodynamic behavior of system in this second stage was unusual, suggesting low membrane-product interaction, which allowed high recirculation flow at low pressures. Using CFS methodology, the AFCo1 were obtained with high efficiency (more than 80 %, taking into consideration the recovery of the proteins). The batches were obtained under aseptic conditions.

### AFCo1 is an efficient adjuvant of Neisseria proteoliposome antigens (PLn)

Table 1 shows that AFCo1 obtained by CFS induced high serum IgG and IgG subclass antibody responses against antigens in the *Neisseria* Proteoliposome (anti-PLn) when administrated by the i.n route. AFCo1 induced significant (p<0.05) IgG2a and IgG1 anti-PLn titers suggesting a preferential induction of a Th1-type response. It was also shown that i.n immunization of mice with AFCo1 induced IgA antibodies in saliva, on day 7 after the last dose. The i.n application of AFCo1 was able to induce measurable levels IgA of anti-PLn in saliva. High levels of IFNγ, but no IL5 productions were also elicited after AFCo1 immunization by the i.n route (Table 1); these results confirm that AFCo1 induce Th1 polarization.

**Table 1:** Immunogenicity AFCo1 obtained by CFS and administered by the i.n route: Anti-PLn IgA, IgG and IgG isotypes measured by ELISA

Groups	IgG(U/mL) sera	IgA(AU/mL) saliva	IgG1(OD) sera	IgG2a(OD) sera	IFNγ(pg/mL)
AFCo1	4415.74*	2147.03*	1.378*	1.62*	2787.04*
Placebo	106.05	101.48	0.102	0.120	162.381
PHA	-	-	-	-	3065.34*

Groups of female BALB/c mice (n = 5) were immunized with AFCo1 by the i.n route (3 doses, 12.5µL in each nostril) with a 7 day interval. Anti-PLn IgA in saliva or serum and anti-PLn IgG and IgG subclass induction by AFCo1 and IFN-γ production by spleen cells from immunized mice. Phosphate Buffer was used for the Placebo group. IFN-γ concentration was evaluated in splenocyte supernatants, 7 days after the last dose and stimulated *in vitro* with PLn or phytohaemagglutinin (PHA). Asterisks denote significant differences between groups * p<0.05.

### Effect of co-administration of AFCo1 with OVA or PepVacTB1 antigens

We also explored the adjuvant properties of AFCo1 obtained following vaccination with OVA or PepVacTB1 antigens. High levels of anti-OVA or anti-PepVacTB1 IgA were detected in saliva when AFCo1 was co-administered with OVA or PepVacTB1 intranasally. The i.n immunized mice with AFCo1+OVA developed significant (p<0.01) amounts of anti-OVA IgG in sera (Table 2). Also significant (p<0.01) amounts of anti-PepVacTB1 IgG in sera were found with i.n co-administration of AFCo1+PepVacTB1 (Table 2). The AFCo1+OVA or PepVacTB1 immunized groups displayed significant (p<0.01) amounts of serum specific IgG1 and IgG2a antibody by the i.n route (Table 2).

**Table 2:** OVA or PepVacTB1specific antibody responses induced by intranasal administration.

	Groups	IgG(OD) sera	IgA(OD) saliva	IgG1(OD) sera	IgG2a(OD) sera
	
I	AFCo1+Ova	0.80**	0.63**	1.5**	0.8274**
	OVA	0.12	0.11	0.10	0.10
II	AFCo1+ PepVacTB1	1.10**	0.45*	1.63**	1.04**
	PepVacTB1	0.2	0.1	0.056	0.105

Results of two independent experiments. Experiment I shows the results of co-administration of AFCo1 with OVA and Experiment II shows the results of co-administration of AFCo1 with PepVacTB1. Female BALB/c mice (n = 5) were inoculated i.n (3 doses with 7 days interval) with AFCo1 (50 µg) admixed with heterologous antigen OVA or PepVacTB1 (20 µg). Control groups were treated with OVA or PepVacTB1 (20 µg per dose per mouse) alone. The data are expressed as the OD means of samples measured at 492 nm. Significant differences between the means of different groups were determined. Asterisks denote significant differences between groups * p<0.05, ** p<0.01

### Preliminary stability studies

The AFCo1 obtained by CFS proved to be stable for a period of 12 months at a temperature of 5 ± 3°C (shelf life) and 40°C (stress). This stability study included serial measurement of size, shape, pH, appearance, protein concentration, preservative concentration, identity, microbial limit, preservative effectiveness and specific anti-PLn IgG and IgA responses in serum and saliva, respectively. All parameters and intervals where established as showed in Table 3. The results of IgA response anti-PLn were not satisfactory at 6-month (4°C) time point, but were satisfactory after 12 months (Table 3). It is possible that processing and successive defrosting of these samples destroyed the IgA. This antibody is very stable, however changes of temperature may unfold IgA structure, therefore protocols to evaluate saliva samples require inhibition of enzymes mucosal fluids with inhibitors or heating sample by short times (56°C by 10 minutes) and store them at -70°C until evaluation [2].

**Table 3:** Results of preliminary stability studies

Description	Limits	2-8^o^C				40^o^C		
		0	3	6	12	1	3	6

**Particle size (light microscopy)**	≥ 60% of under 50 µm	S	-	S	S	NP	NP	NP

**Shape (light microscopy)**	elongated tubular structures	S	-	-	S	NP	NP	NP

**pH**	7±0.5	7,4	7,1	7,1	7,1	7,2	7,2	7,3

**Appearance**	Milky suspension	S	S	S	S	S	S	S

**Free Ca2+ (atomic absorption)**	<5 mM	<0,1	<0,1	NP	<0,1	-	<0,1	<0,3

**Protein concentration (Lowry)**	1,2±0,2 mg/mL	1,19	1,26	NP	1,27	1,19	1,2	1,26

**Preservative (atomic absorption)**	0.07-0.13 mg/mL	0.1	NP	NP	0,09	NP	NP	NP

**Identity (SDS/PAGE)**	two majority bands between 36 and 46 kDa and two minor between 70 and 85 kDa	P	P	NP	P	P	P	P

**Microbial limit**	Less than <10^2^ aerobic bacteria or fungi by *Pseudomonas aeruginosa*m. Absence of *Staphylococcus aureus*, *Escherichia coli* and Salmonella	S	S	NP	NP	NP	NP	NP

**Preservative**	Microbial growth reduction <2 log at 14 days and no microbial growth increased from 14 to 28 days	S	S	NP	S	S	S	S

**Potency ELISA(IgA)**	IgA in saliva anti OMV > 250 UA	261.33	503	NS	882.17	NS	NS	NS

**Potency ELISA(IgG)**	IgG in serum anti OMV	S	S	S	S	S	S	S

Legend: S, Satisfactory; NS, Not satisfactory; NP, Not planned; – , Not evaluated or planned; P, Positive

## Discussion

Previous studies have demonstrated the production of AFCo1 on a lab scale and preliminary work at higher scale demonstrated that CFS could be used to obtain larger amounts of cochleates [4].

In the present work, the efficiency of the immune response of the AFCo1 obtained by tangential diafiltration (pilot scale) was demonstrated. The results suggest that the i.n route is able to induce significant and antigen specific IgA levels in saliva and of IgG in serum after immunization of BALB/c mice with 3 dose of AFCo1. High titers of IgG1 and IgG2a in serum were also induced with i.n administration of AFCo1. The i.n administration with AFCo1 generated a Th1 polarized pattern, characterized by high levels of IgG2a and induction of IFNγ. This agrees with several reports that suggest that nasal associated lymphoid tissue is a place where the induction of Th1 response is favored [7]. In addition, the immunogenicity and pattern induction of AFCo1 obtained by CFS corresponds with previous results obtained with AFCo1 obtained by the lab scale dialysis rotary method [2,8].

In this study we also evaluated the capability of splenocytes obtained from the immunized animals to be stimulated *in vitro* to produce IFNγ. The AFCo1 efficiently stimulate this cytokine suggesting activation and differentiation of the CD4 lymphocytes (LΦT) into Th1 cells. Considering that this cytokine is produced principally by the LΦT, then IFNγ might be produced by Th1 type cells isolated from spleen [9].

We explored the adjuvant properties of AFCo1 with addmixed heterologous antigens (OVA or PepVacTB1) via the i.n route. The results showed that i.n immnunization with AFCo1+OVA or AFCo1+PepVacTB1 induced high levels of anti OVA or PepVacTB1 IgG, IgG1 and IgG2a antibodies in sera and specific IgA in saliva. We have demonstrated the adjuvant capability of the AFCo1 obtained by CFS when co-administered with heterologous antigens by the i.n route. Previous studies have shown i.n immunization with AFCo1 obtained by the dialysis method has adjuvant effects on co-administered heterologous antigens such as OVA [8].

In addition, stability studies of AFCo1 were satisfactorily concluded in order to develop safe formulations for nasal application. Pharmaceutical and preclinical evaluation of new adjuvants, as part of vaccine formulations, is relevant in their efficacy and safety evaluation. Detailed information about the preclinical experiments with AFCo1 were previously published by Infante *et al.*[10]. These authors demonstrate that AFCo1 successfully passed the tests of local tolerance and preclinical toxicity (single and repeated doses). A single dose was classified as non irritating locally and showed no systemic toxicity, so AFCo1 is considered potentially non toxic for intranasal administration to humans in this experiments. Repeated doses experiments, which included: complete blood count, biochemical and histopathological tests, showed that AFCo1 was rated as non-irritating locally and with no evidence of systemic toxicity, so it was considered potentially non toxic to humans by intranasal administration in repeated doses [10].

New vaccines containing new adjuvants represent a challenge for regulatory authorities, which are actively discussing standardization of guidelines to evaluate and develop adjuvants in the near future [11].

In summary, the present study demonstrates the efficacy of AFCo1 obtained on pilot scale through the crossflow system. AFCo1 obtained by this methodology produced high level of specific antibodies in serum and saliva by the mucosal route and have the capacity to enhance the mucosal response against *Neisseria* antigens in AFCo1 using OVA or PepVacTB1 as model antigens. Tangential filtration would ensure the production of a large scale adjuvant that is safe and immunogenic.

## Competing interests

The authors declare that they have no competing financial interests.

## Authors' contributions

CZ conceived of the study, participated in its design, AFCo1 production, discussion of results and drafted the manuscript; DG participated in the AFCo1 production; RA participated in AFCo1 production and manuscript draft; JC participated in the evaluation of immune response; ML participated in the evaluation of results of stability; EG participated in the evaluation of immune response and animal work; BR participated in the evaluation of immune response; MC participated in the analytical evaluation of AFCo1; JB participated in the analytical evaluation of AFCo1; OC participated in the analytical procedures; LG conceived the evaluation of adjuvant potential of AFCo1 with PepVacTB1 antigens, participated in its design and discussion of results; OP conceived of the study, and participated in its design and discussion of results; All authors have read and approved the final manuscript.

## Declarations

This article has been published as part of *BMC Immunology* Volume 14 Supplement 1, 2013: Proceedings from Delivery Systems and Current strategies to drug design. The full contents of the supplement are available online at http://www.biomedcentral.com/bmcimmunol/supplements/14/S1
